# J. L. Meunier

**Published:** 1911-09-30

**Authors:** 


The death is announced, at the age of fifty-one, of Dr. J. L. Meunier, senior physician to the Hôtel Dieu of Pontoise. He was a well-known writer on medical history, frequently contributing to the *Bulletin de la Société Francaise d'Histoire de la Médecine* and to *Janus*. Last year he published an important work entitled "Histoire de la Médecine" which met with well-deserved recognition.

